# Can conditional cash transfers improve the uptake of nutrition interventions and household food security? Evidence from Odisha’s *Mamata* scheme

**DOI:** 10.1371/journal.pone.0188952

**Published:** 2017-12-11

**Authors:** Kalyani Raghunathan, Suman Chakrabarti, Rasmi Avula, Sunny S. Kim

**Affiliations:** 1 Poverty, Health and Nutrition Division, International Food Policy Research Institute (IFPRI), New Delhi, India; 2 Poverty, Health and Nutrition Division, International Food Policy Research Institute (IFPRI), Washington D.C., United States of America; University of Waterloo, CANADA

## Abstract

There is considerable global evidence on the effectiveness of cash transfers in improving health and nutrition outcomes; however, the evidence from South Asia, particularly India, is limited. In the context of India where more than a third of children are undernourished, and where there is considerable under-utilization of health and nutrition interventions, it is opportune to investigate the impact of cash transfer programs on the use of interventions. We study one conditional cash transfer program, *Mamata scheme*, implemented in the state of Odisha, in India that targeted pregnant and lactating women. Using survey data on 1161 households from three districts in the state of Odisha, we examine the effect of the scheme on eight outcomes: 1) pregnancy registration; 2) receipt of antenatal services; 3) receipt of iron and folic acid (IFA) tablets; 4) exposure to counseling during pregnancy; 5) exposure to postnatal counseling; 6) exclusive breastfeeding; 7) full immunization; and 8) household food security. We conduct regression analyses and correct for endogeneity using nearest-neighbor matching and inverse-probability weighting models. We find that the receipt of payments from the *Mamata* scheme is associated with a 5 percentage point (pp) increase in the likelihood of receiving antenatal services, a 10 pp increase in the likelihood of receiving IFA tablets, and a decline of 0.84 on the Household Food Insecurity Access Scale. These results provide the first quantitative estimates of effects associated with the *Mamata* scheme, which can inform the design of government policies related to conditional cash transfers.

## Introduction

Despite rapid declines in child undernutrition in the last decade in India, 38% of children under 5 years are stunted, 35% are underweight and 21% are wasted [1]. The coverage of nutrition interventions varies widely. While some interventions such as institutional delivery (79%), full immunization (62%), and vitamin A supplementation (60%) have near universal coverage nationally, others such as consumption of IFA tablets during pregnancy (30%), receiving four or more antenatal care (ANC) visits (51%) [1] and receipt of food supplements from pregnancy through early childhood (<50%) have far lower coverage [2]. In addition, considerable geographical heterogeneities exist both across states, and across lower administrative levels within states.

India has in place various programs and policies to improve child nutrition outcomes. The Integrated Child Development Services (ICDS) and the National Health Mission (NHM) are two national programs that deliver all of the essential nutrition interventions (ENIs) through their cadres of frontline workers called Anganwadi Workers (AWWs) and Accredited Social Health Activists (ASHAs) respectively [3]. However, the quality of service delivery has been sub-par [4–7]. Furthermore, there is low awareness of the interventions offered, with only 9.5% of mothers of children aged 0–6 months and 8.3% of currently pregnant women aware of all 6 services provided through the ICDS [2]. With the understanding that achieving 90% coverage of the ENIs can contribute to 20% reduction in stunting [8], it is imperative that investments are made to improve the service delivery and uptake of interventions.

Over recent years there has been an increasing interest in the use of cash transfer programs to improve nutrition and health outcomes. There is considerable global evidence on the impact of the cash transfer programs—conditional or unconditional—on food security, dietary diversity, utilization of healthcare services, child cognitive development, and on morbidity, anemia, and anthropometry for both mother and child [9–23], but evidence in the Indian context is limited. As part of the 2017 Union Budget, the Indian Finance Minister announced a five-fold increase in the budget allocation to the existing Maternity Benefits Program. This amounts to an increase in the allocation from USD 60 million in 2016–17 to USD 770 million in 2017–18. Though CCTs have been part of the Indian policy landscape since the early 1990s (for example, the *Muthulakshmi Reddy Maternity Benefit* (MRMB) scheme and the Girl Child Protection Scheme in Tamil Nadu), these recent increases in the amounts being disbursed under pre-existing CCT programs, as well as the introduction of new CCTs at national and state-levels, have reopened the debate about their effectiveness. At present, much of the India-specific evidence on cash transfers comes from aa national CCT program, the *Janani Suraksha Yojana (JSY)*, introduced in 2005 and designed to promote institutional deliveries. It has been shown to increase medically-supervised births [24–26] and the likelihood of receiving ANC [25], although coverage is low and inconsistent with poor targeting and service quality [25,27–29].

While there is evidence that conditioning financial incentives on the utilization of healthcare services can have a significant and positive impact on the demand for and supply of these services [10,21–23,30–32], care should be taken in designing a CCT. A large number of complex conditions that rely heavily on the availability of services can result in low compliance, both because of the time and effort required to meet conditions, as well as the lack of awareness around the requirements [28,33]. Conditions can also create exclusions, since poorer and more marginalized households may find it harder to access services [34,35].

To understand the effect of cash transfers on the delivery and uptake of the nutrition interventions and to build the evidence base for India on the impact of cash transfer programs, we examined the effect of a CCT program in the eastern-Indian state of Odisha, known as the *Mamata* scheme.

**Fig 1:**
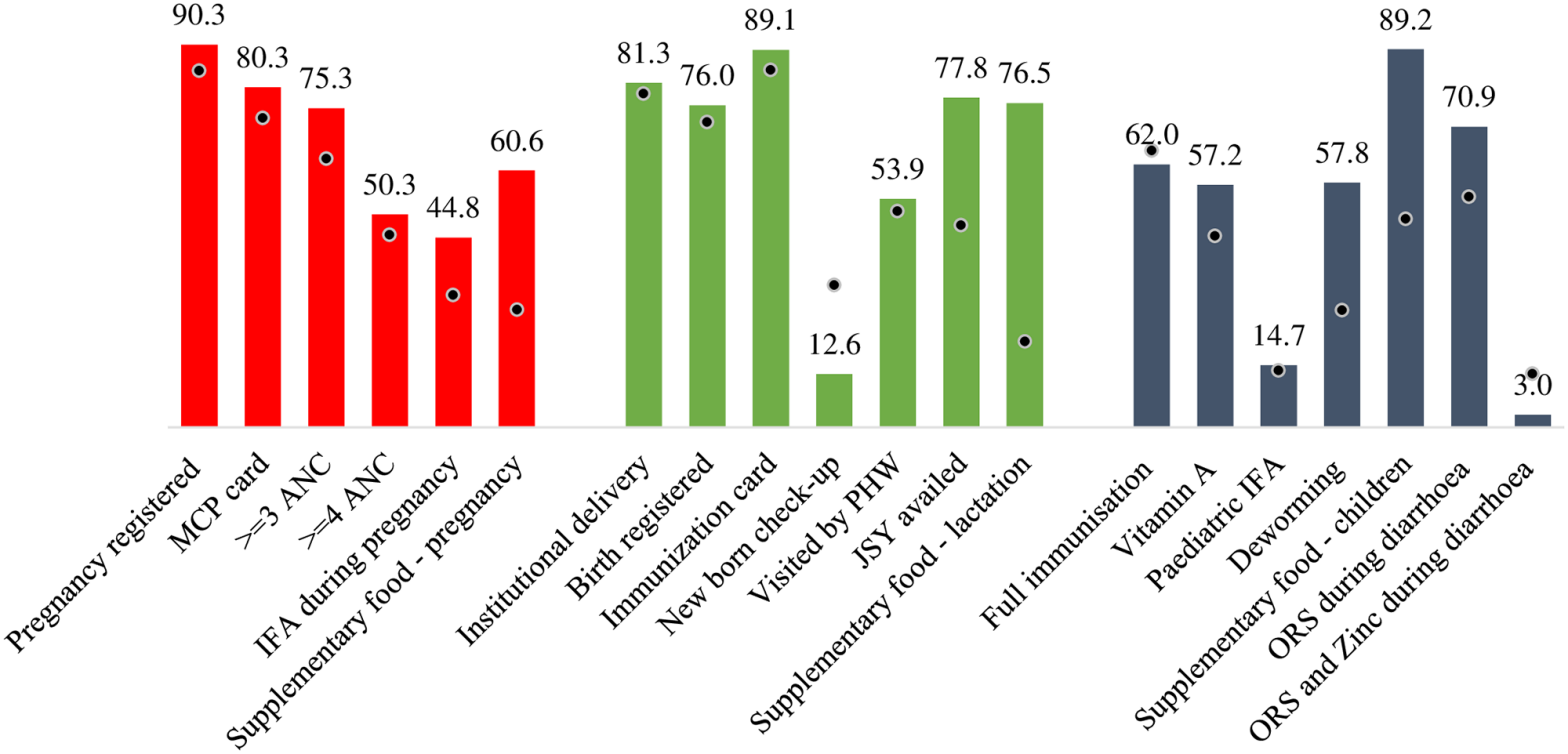
Coverage of nutrition interventions across the continuum of care in Odisha. The data represented here is from the Rapid Survey of Children (RSOC 2014). Columns represent the coverage of the indicators in Odisha in 2014. Dots represent the national average of the indicators in 2014. Coverage is reported in percentages. Left-most bars in red denote interventions delivered during pregnancy; central bars in green denote those delivered during the first 6 months post-partum; right-most bars in blue are those delivered from 6 months to 5 years of age. MCP = Mother child protection; ANC = Antenatal care visit, IFA = Iron-folic acid, PHW = Public/primary health worker; JSY = Janani Suraksha Yojana (A cash transfer to promote institutional delivery); ORS = Oral dehydration salts.

Odisha is economically poor but has a stronger and more inclusive healthcare system than many other more well-off parts of India [36], and seems to be delivering on health and nutrition. The state has made significant improvements in its service delivery over the last two decades, and currently performs better than the national average on the coverage of several health and nutrition interventions (Fig 1). Much of this has been attributed to the strengthening of the health system through various government policies and initiatives [37], including the introduction of the *Mamata* scheme and other entitlement schemes [36].

The *Mamata* scheme was launched in September 2011 by the Women and Child Department of the Government of Odisha with the aim of providing partial wage compensation to pregnant and nursing mothers, improving the utilization of health services, and improving infant and young child feeding practices. The government of Odisha stipulates that all women over the age of 19 years with up to two live births are eligible for this transfer, provided they meet the conditions. The scheme transfers a total of 5000 INR (approximately USD 75) directly to the beneficiary’s bank account through four installments on fulfilment of certain conditions (Table 1)–payable at the end of the second trimester, and at 3, 6, and 9 months after delivery. In the state with the third lowest average annual household income in the country [38], this monetary transfer contributes to 6% of the average household annual income.

**Table 1:** Installments, conditions and means of verification for the *Mamata* scheme (reproduced from [39]).

**Instalment 1: end of the second trimester (INR 1500)**	**Instalment 2: 3 months after delivery (INR 1500)**
Conditions: i. Pregnancy registered at the AWC/Mini AWC. ii. Received at least one antenatal check-up (out of optimal 3). iii. Received IFA tablets. iv. Received at least one TT vaccination (out of optimal 2). v. Received at least one counselling session at the AWC/ Village Health and Nutrition Day (VHND). Means of verification: i. MCP card ii. Scheme register	Conditions: i. Child birth is registered. ii. Child has received BCG vaccination. iii. Child has received Polio 1 and DPT-1 vaccination. iv. Child has received Polio 2 and DPT-2 vaccination. v. Child has been weighed at least two times after birth (out of optimal 4 times including at birth). vi. After delivery, mother has attended at least two IYCF counselling sessions at the AWC / VHND / Home Visit (out of optimal 3 times), as certified by the AWW. Means of verification: i. MCP card ii. Scheme register
**Instalment 3: after the infant completes 6 months (INR 1000)**	**Instalment 4: after the infant completes 9 months (INR 1000)**
Conditions: i. Child has been exclusively breastfed for first six months. ii. Child has been introduced to complementary foods on completion of six months. iii. Child has received Polio 3 and DPT-3 vaccination. iv. Child has been weighed at least two times between age 3 and 6 months (out of optimal 3). v. Mother has attended at least two IYCF counselling sessions between 3 and 6 months of lactation, at the AWC/VHND/Home Visit (out of optimal 3). Means of verification: i. MCP card ii. Scheme register ii. Self-certification on MCP card	Conditions: i. Measles vaccine has been given before the child is one year old. ii. Vitamin A first dose has been given before the child is one year old. iii. Age-appropriate complementary feeding has started and is continuing. iv. Child is weighed at least two times between 6 months to 9 months Means of verification: i. MCP card ii. Scheme register iii. Self-certification on MCP card

Source: Ministry of Women and Child Development, Government of Odisha. Notes: AWC = Anganwadi Centre, AWW = Anganwadi Worker, IFA = iron folic acid, TT = tetanus toxoid, VHND = village health and nutrition day, MCP = mother child protection, BCG = Bacillus, Calmette and Guerin, DPT = diphtheria, pertusis (whooping cough), and tetanus, IYCF = infant and young child feeding.

If the *Mamata* scheme is effective in increasing adherence to the conditions which have a direct bearing on maternal and child health and contribute to household income, then it could be showcased as a model CCT. This study provides the first quantitative estimates of effects associated with the *Mamata* scheme on the exposure to nutrition interventions, and on household food security. In doing so, we contribute to the limited body of evidence on CCTs in the Indian context.

### Ethical approval

Ethical clearance for this research was provided by IFPRI’s Institutional Review Board. The study was also approved by the Women and Child Development Department and the Department of Health and Family Welfare in India. For household interviews, a full informed consent process was followed, including verbal consent in the presence of the witness in situations where the respondent was not literate. Where verbal consent was obtained, the consent form was used, but marked to note that the type of consent obtained was verbal in the presence of a witness. Our ethics committee approved this method of obtaining consent.

## Materials and methods

### Description of the dataset

The household survey data used in this paper was collected as part of a larger mixed-methods observational study, the purpose of which was to examine the delivery of a set of ENIs by the ICDS and the NHM [40]. No interventions were administered through this study, and only the condition of current operations was assessed.

#### Study sample

We conducted a study in three districts of Odisha, to examine the state of delivery and use of ten select ENIs and the role of inter-sectoral coordination in service delivery. Data collection was conducted from February to March 2014. The study covered several critical nutrition interventions across the continuum of care (pregnancy to 2 years of age), ranging from interpersonal counseling to micronutrient supplementation and provision of supplementary food. The three districts were selected from among the 30 total districts in the state. Existing district-level survey data (IIPS 2007, 2010) were used to construct a set of criteria pertaining to service coverage and household factors (e.g., coverage of immunization and vitamin A supplementation, three or more ANC visits, and institutional delivery; access to toilet and electricity; and type of cooking fuel), and to examine the changes in these indicators between two survey rounds. All districts were grouped into three categories: better-performing districts (i.e., those with positive change over time), average performing districts (i.e., no change), and poorly performing districts (i.e., those with negative change). Then, one district from each category was randomly selected: Jagatsinghpur, better performing; Keonjhar, average performing; and Kalahandi, poorly performing. In each district, we randomly selected four blocks (n = 12) and 25 villages (n = 300) from each block. Four households (two with children 0–5.9 months of age and two with children 6.0–23.9 months of age) were selected randomly from the list of households at the Anganwadi Centre (AWC) in each village. Respondents were mothers of these children from the selected households. After data cleaning, our final sample size was 1161 households.

#### Outcome measures

The outcomes of interest are:

1. Pregnancy registration: a dummy for the respondent having registered her pregnancy with the AWW.
2. Receipt of ANC services: a dummy for the respondent having received any antenatal services or counseling (from any source).
3. Receipt of IFA tablets from the AWW/ ASHA: a dummy for the respondent having received IFA tablets during pregnancy from either the AWW or the ASHA.
4. Exposure to counseling during pregnancy: A dummy for the respondent having received counselling on breastfeeding during pregnancy (from any source).
5. Exposure to postnatal counseling on the duration of breastfeeding: dummy for the respondent having received postnatal counselling on the duration of exclusive breastfeeding (from any source).
6. Exclusive breastfeeding: a dummy for the index child having been exclusively breastfed for the first six months (self-reported by the mother).
7. Full immunization: a dummy for a child between the ages of 1 and 2 years being fully immunized, i.e. having received BCG, Polio 1, 2 and 3, DPT-1, 2 and 3 and measles vaccination.
8. Household food security: To measure household food insecurity we use the Household Food Insecurity Access Score (HFIAS) [41]. This set of indicators and accompanying domains is constructed from 9 incidence-related and 9 frequency-related questions around the experience of food insecurity. These questions are used to construct an overall HFIAS score for the household that ranges from 1 to 27, with higher values indicating greater food insecurity, 3 domains of insecurity–anxiety, insufficient quality, and insufficient quantity, and 9 indicators for the various measures of food insecurity. We examined the overall HFIAS score (1–27), whether the household experienced each of the 9 HFIAS conditions, and whether the household experienced each of the 3 HFIAS domains.

These outcome measures were selected for corresponding to the *Mamata* conditions from Table 1. In keeping with the timing of the scheme payments, the impact of *Mamata* money on the first four outcomes is estimated for all children in the sample, on the fifth outcome only for the sample of children above the age of 3 months, on the sixth outcome for children above the age of 6 months, and on the seventh outcome only for children above the age of 12 months.

The exclusive breastfeeding variable is self-reported by the mother, and is hence subject both to social desirability bias and over-reporting in order to continue receiving scheme benefits [23,42]. However, since the receipt of the third installment of money is based on similar self-certification by the mother on the Mother-Child Protection (MCP) card, we retain this measure with the caveat that it may not reflect accurately the rates of exclusive breastfeeding in the sample.

#### Covariates

In all the regression analyses, we control for individual, household and geographic characteristics, which include:

*Individual-level characteristics*: Maternal age, maternal education level (no education, primary education (grades 1 to 5), middle school (grades 6 to 9), completed class 10, completed class 12, or college and higher), maternal caste (Schedule Tribe (ST), Other Backward Classes (OBC) and General caste, and Scheduled Castes (SC)), and paternal education in years.

For the exposure to nutrition interventions, we also control for child age, child sex, and whether the mother ever participated in the immunization day (also known as Village Health and Nutrition Day (VHND)). The VHND is held once a month at the village healthcare center and delivers several ENIs such as the distribution of food supplements, counseling on infant and young child feeding (IYCF), and growth monitoring.

*Household-level characteristics*: Household size and socio-economic status (SES). SES is constructed using factor analysis using information on the number of durable assets the household owns (items like a refrigerator, mattress, bicycle, water pump, etc.), ownership of the house and the land on which it is built, ownership of other land, and ownership of livestock (cattle, buffalo, goats, chickens, pigs). The full list of assets is in S1 Table.

For the analysis on household food security, we also control for any purchase from the Public Distribution System (PDS). The PDS is a national food security program that provides a certain quantity of food and non-food items at subsidized prices to poor households every month. The standard commodities available for sale through the PDS are rice, wheat, coarse cereals, sugar and kerosene. PDS cards are issued to poor households with particular attention to inclusion of the poorest of the poor and most vulnerable sections of society such as landless agricultural laborers, marginal farmers, rural artisans/craftsmen (e.g. potters, weavers, blacksmiths, carpenters) and urban informal-sector workers (e.g. potters, rickshaw-pullers, cart-pullers, fruit and flower sellers on the pavement). The village governance institutions–the *Gram Panchayats* and *Gram Sabhas*—are involved in the identification of eligible families.

*Geographic characteristics*: District- and block-level fixed effects.

### Statistical methods

We examined the effects of receiving money from the *Mamata* scheme on the exposure to ENIs and household food security.

First, we studied the unadjusted bivariate associations between the receipt of *Mamata* money and adherence to the conditions of the scheme for children of the appropriate age-group. To do so, we regressed the binary outcome variable on whether or not the respondent received *Mamata* money. Standard errors were clustered at the village level.

Then we conducted linear probability regression models, estimated by ordinary least squares (OLS). For child *i* belonging to household *h* in village *v* in block *b* in district *d*, we estimated

$$Y_{ihvbd}=\alpha +X_{ihvbd}\beta +Z_{hvbd}\gamma +\;\delta_{b}+\mu_{d}+\epsilon_{ihvbd,}$$

where *Y*_*ihvbd*_ is the child-level outcome of interest (e.g. whether the pregnancy was registered or whether the child received certain vaccinations), *X*_*ihvbd*_ is a vector of child-specific covariates, such as child age and sex, *Z*_*hvbd*_ is a vector of parental and household-level covariates, such as maternal age, education and caste, paternal education, and household SES and size. *δ*_*b*_ and *μ*_*d*_ are (respectively) block- and district-level fixed effects. Finally, *ϵ*_*ihvbd*_ is the child-specific error term. Standard errors were clustered at the level of the village, since this is the geographical level where government health workers like the AWW and the ASHA operate and where we would expect similarities in health service quality and access. Similar equations at the household level were used to assess the impact of *Mamata* scheme on household food security. Here we did not control for child age and sex, but instead controlled for use of the government subsidized-food entitlement scheme, the PDS.

#### Alternative specifications

While the results based on the OLS regression are informative, given the cross-sectional nature of the dataset we cannot correct for endogeneity. Specifically, unobserved factors in the error term *ϵ*_*ihvbd*_ could be correlated both with receiving money from the *Mamata* scheme as well as with the outcomes of interest. For example, more motivated mothers may be more likely to enroll in the scheme, but even without the presence of the scheme they may be more likely to invest in the health and nutrition of their children. We used two methods to correct for this endogeneity.

The first method used was nearest-neighbor matching. We matched each unit in the treatment group (those who have received some money from the *Mamata scheme*) to 5 units in the control group (those who did not receive any money from the *Mamata* scheme) that were the ‘closest’ to the treated unit. This ‘closeness’ was achieved by matching units along a vector of pre-determined characteristics—i.e. those that are not likely to be affected by *Mamata* scheme money, or determined by the same unobserved factors as the receipt of this money—and employing a distance metric (in our case, Euclidean) that minimized the distance between these observations. We used maternal age, caste and education, paternal education, household size and SES, and geographic characteristics to match units. The graphical depiction of the common support assumption is presented in S1 Fig.

The second method used was inverse-probability weighting (IPW). This method uses the inverse of the probability of being in the observed treatment group in order to correct the average treatment effect estimates. The probabilities are calculated by fitting a model of treatment status on a set of characteristics. We used a probit model to calculate the probabilities, including the same set of characteristics as in the OLS models.

## Results

Nearly 60% of the mothers in our study had enrolled in the scheme, and over 90%of those enrolled reported receiving money from the *Mamata* scheme (Table 1). There was, however, some heterogeneity across the 3 districts of Odisha in our sample in the proportion of women who received any money from the scheme. Jagatsinghpur had the highest proportion of beneficiaries, with 73.9% of the women receiving at least one installment, while only 46.3% in Kalahandi and 42.3% in Keonjhar received at least one installment.

While the average amount received appeared to increase monotonically with child age, the transfers were smaller than expected from the specifications of the scheme, suggesting delays in payments to beneficiaries or the deposit of incorrect amounts of money. A comparison of payment timing by tranche with amounts actually received (Tables 1 and 2), indicated that mothers receive 900 and 1300 INR less, on average, than the expected amount at 3 and 6 months after delivery, respectively. This suggests that insufficient deposits or delays in transfers typically occurred around the second and third tranches. However, by the time the child reached 9 months of age, mothers reported receiving 4500 INR, on average, which is 500 INR less than the full intended transfer amount. Among those women who received any money from the scheme, the most commonly reported uses of the money were household savings and expenditure on food (27.9%), and expenditure on child health, food and care (25.6%) (Table 2).

**Table 2:** Exposure to the *Mamata* scheme and reported use of money received.

Respondent women	Mean (SD)/ (%)
Enrolled in *Mamata* scheme (N = 1161)	58.5
Received any money from *Mamata* scheme (N = 1161)	54.0
Proportion of women who received any money from *Mamata* in:	
Jagatsinghpur (N = 379)	73.9
Kalahandi (N = 385)	46.3
Keonjhar (N = 397)	42.3
Amount of money received by child age (among those who received money ^1^) (in INR ^2^):	
Child age < = 3 mo (N = 138)	1874.6 (954.4)
Child age >3 & < = 6 mo (N = 130)	2183.1 (971.2)
Child age >6 & < = 9 mo (N = 80)	2743.8 (1087.9)
Child age >9 mo (N = 274)	4502.2 (870.7)
All (N = 622)	3208.4 (1504.2)
Reported use of *Mamata* money ^3^ (N = 523):	
Household savings and expenditure on food	27.9
Expenditure on child health, food and care	25.6
Household expenditure on medicines and medical checkups	15.1
Expenditure on maternal health, care and nutrition (including medicines for the mother)	13.2
Bank deposits in the child’s name	4.6
Other	13.6

^1^. 5 respondents who reported receiving money did not report the amount received.

^2^. INR = Indian Rupees.

^3^. If the food was not specifically mentioned as being purchased for either the mother or the child, then it is included in household expenditure. This category also includes savings/deposits (which do not explicitly mention the child), other household expenses, etc. Similarly, if the medicines and medical treatment recipient is not explicitly mentioned, it is included under household expenditure on medicines and medical checkups. 104 respondents who reported receiving money did not report what they used the money for.

### Characteristics of women receiving money from the *Mamata* scheme

In our sample, women who received any money from the scheme were 1.5 years younger than women who did not, and their children were 2 months older than those of non-beneficiary women (Table 3). The scheme did not appear to successfully target households from lower SES strata. A lower proportion of *Mamata* beneficiaries were from the lowest SES quintile (12%) compared to the highest (26.6%). A similar pattern is observed by educational status, where only 12% of beneficiaries had no education, compared to 30% among non-beneficiaries. Also, only a small fraction of beneficiary women belonged to the scheduled tribes’ category (17%), which is among the most marginalized groups in the country.

**Table 3:** Sample characteristics by treatment status.

		Among those who did not receive any *Mamata* money (N = 534)	Among those who received *Mamata* money (N = 627)	All (N = 1161)	p-value
		Mean (SD)/ % [lb, ub]	Mean (SD)/ % [lb, ub]	Mean (SD)/ % [lb, ub]	
	Maternal age in years	26.7 (4.8)	25.1 (3.7)	25.8 (4.3)	0.000
	Paternal education	9.6 (5.8)	10.1 (4.9)	9.8 (5.4)	0.107
	Household size	5.5 (1.5)	5.5 (2.0)	5.5 (1.8)	0.962
	Child's age in months	7.5 (6.6)	9.6 (6.7)	8.7 (6.8)	0.000
	Child is a girl	46.3 [42, 50.5]	53.3 [49.4, 57.2]	50.0 [47.2, 52.9]	0.017
Household SES (assets, livestock, land, toilet)	Wealth quintile 1	29.8 [25.9, 33.7]	12.0 [9.4, 14.5]	20.2 [17.8, 22.5]	0.000
	Wealth quintile 2	22.7 [19.1, 26.2]	17.7 [14.7, 20.7]	20.0 [17.7, 22.3]	0.035
	Wealth quintile 3	18.5 [15.2, 21.8]	21.1 [17.9, 24.3]	19.9 [17.6, 22.2]	0.285
	Wealth quintile 4	17.2 [14, 20.4]	22.7 [19.4, 25.9]	20.2 [17.8, 22.5]	0.022
	Wealth quintile 5	11.8 [9.1, 14.5]	26.6 [23.2, 30.1]	19.8 [17.5, 22.1]	0.000
Maternal education	No education	30.0 [26.1, 33.9]	12.3 [9.7, 14.9]	20.4 [18.1, 22.7]	0.000
	Primary school (1–5)	20.8 [17.3, 24.2]	14.0 [11.3, 16.8]	17.1 [15, 19.3]	0.002
	Middle school (6–9)	28.3 [24.4, 32.1]	39.9 [36, 43.7]	34.5 [31.8, 37.3]	0.000
	Completed class 10	11.8 [9.1, 14.5]	22.2 [18.9, 25.4]	17.4 [15.2, 19.6]	0.000
	Completed class 12	3.8 [2.1, 5.4]	6.1 [4.2, 7.9]	5.0 [3.7, 6.3]	0.071
	College and higher	5.2 [3.3, 7.1]	5.6 [3.8, 7.4]	5.4 [4.1, 6.7]	0.800
Maternal caste category	Scheduled caste	13.7 [10.7, 16.6]	21.1 [17.9, 24.3]	17.7 [15.5, 19.9]	0.001
	Scheduled Tribe	39.1 [35, 43.3]	17.4 [14.4, 20.4]	27.4 [24.8, 30]	0.000
	Other backward classes	33.7 [29.7, 37.7]	44.0 [40.1, 47.9]	39.3 [36.5, 42.1]	0.000
	General	12.0 [9.2, 14.7]	17.2 [14.3, 20.2]	14.8 [12.8, 16.9]	0.012

### Unadjusted outcomes by treatment status

Our unadjusted analysis showed that adherence to the conditions of the scheme was higher among women who received any money from the scheme, as compared to non-beneficiary women (Table 4). Specifically, beneficiaries were more likely to receive IFA tablets and counseling during pregnancy, and the recommended doses of vaccinations including BCG, Polio and DPT for their children (unadjusted p<0.01). However, there were no statistically significant differences in the proportion of women who received postnatal counseling or practiced exclusive breastfeeding for six months.

**Table 4:** Adherence to *Mamata* conditions by treatment status (unadjusted).

	*Mamata* conditions	Have not received any money from *Mamata*		Have received at least one installment		All		p-value
		%	N	%	N	%	N	
**Condition set 1: completion of 6 months of pregnancy**	Pregnancy registered at the AWC	99.1	534	100.0	627	99.6	1161	0.059
	Received at least one antenatal checkup	92.7	534	99.2	627	96.2	1161	0.000
	Received IFA tablets	72.5	534	84.5	627	79.0	1161	0.000
	Received at least one counselling session while pregnant	71.4	534	79.1	627	75.5	1161	0.009
**Condition set 2: completion of 3 months post-delivery**	Received at least one post-delivery counselling session	49.0	390	51.4	537	50.4	927	0.127
	Child received BCG vaccine	97.7	390	99.6	537	98.8	927	0.015
	Child received Polio1 vaccine	95.1	390	99.4	537	97.6	927	0.000
	Child received Polio2 vaccine	88.5	390	96.1	537	92.9	927	0.000
	Child received DPT1 vaccine	94.6	390	98.9	537	97.1	927	0.000
	Child received DPT2 vaccine	91.0	390	95.9	537	93.9	927	0.000
**Condition set 3: completion of 6 months post-delivery**	Child received Polio3 vaccine	85.1	241	92.5	384	89.6	625	0.000
	Child received DPT3 vaccine	86.7	241	92.7	384	90.4	625	0.000
	Child exclusively breastfed for 6 months	86.4	228	85.9	375	86.1	603	0.8564
**Condition set 4: completion of 9 months post-delivery**	Child received measles vaccine	87.0	185	92.2	306	90.2	491	0.001
	Child received Vitamin A	81.1	185	88.9	306	86.0	491	0.001

Numbers shown here are unadjusted proportions across the treatment and control groups.

The prevalence of food insecurity was lower among beneficiary women compared to non-beneficiaries (Table 5). Smaller proportions of *Mamata* beneficiaries reported experiencing food insecurity on specific measures such as anxiety, insufficient quality and quantity (unadjusted p<0.01).

**Table 5:** Household food insecurity measures by treatment status (unadjusted).

	Have not received any money from *Mamata* (N = 534)		Have received at least one installment (N = 627)		All (N = 1161)		p-value
	Mean/ Proportion	SD	Mean/ Proportion	SD	Mean/ Proportion	SD	
HFIAS score (1–27, higher score indicates greater insecurity)	3.89	5.59	2.93	4.12	3.44	4.98	0.001
*HFIAS conditions*:							
Worried that household wouldn't have enough food	39.89		28.87		33.94		0.000
Not able to eat preferred foods	45.13		36.52		40.48		0.013
Had to eat only a few varieties of food	34.27		19.94		26.53		0.000
Ate food that didn't want to eat	32.02		19.14		25.06		0.000
Ate smaller meals	31.27		16.27		23.17		0.000
Ate fewer meals	26.78		13.56		19.64		0.000
No food in house	19.29		9.73		14.13		0.000
Slept hungry at night	17.04		8.13		12.23		0.000
Did not eat for 24 hours	15.73		7.50		11.28		0.000
*HFIAS domains*:							
Anxiety	39.89		28.87		33.94		0.000
Insufficient quality	55.43		43.22		48.84		0.003
Insufficient quantity	38.58		23.13		30.23		0.000

Numbers shown here are unadjusted proportions across the treatment and control groups; HFIAS = Household Food Insecurity Access Score.

### OLS regression results

Table 6 presents the OLS regression results for the exposure to nutrition interventions and the overall HFIAS score. The full set of results for the 9 sub-components and 3 domains of the HFIAS is in S2 Table.

**Table 6:** OLS regression results on exposure to nutrition interventions and household food security.

	Registered pregnancy	Received any antenatal service	Received IFA tablets during pregnancy	Received counselling during pregnancy	Received counselling on duration of breastfeeding	Child was exclusively breastfed	Child is fully immunized	HFIAS score (1–27)
Received money from *Mamata*	0.01*	0.05***	0.10***	0.01	-0.04	0.00	0.06	-0.84**
	(0.00)	(0.02)	(0.03)	(0.03)	(0.04)	(0.03)	(0.05)	(0.36)
Households bought from PDS in the last month								-1.23***
								(0.36)
Participated in VHND	0.00	0.03**	0.05*	0.15***	-0.00	0.06*	0.06	
	(0.00)	(0.02)	(0.03)	(0.03)	(0.04)	(0.04)	(0.04)	
Maternal age	0.00	0.00	0.00	0.00	0.00	0.00	0.00	-0.02
	(0.00)	(0.00)	(0.00)	(0.00)	(0.00)	(0.00)	(0.00)	(0.04)
**Maternal education**:								
No education (ref)	-	-	-	-	-	-	-	-
Primary school	-0.01	0.01	-0.10**	-0.07	0.10*	0.12***	-0.04	0.18
	(0.00)	(0.03)	(0.04)	(0.05)	(0.05)	(0.04)	(0.08)	(0.58)
Middle school	-0.01*	0.03*	-0.11***	-0.01	0.13**	0.05	0.00	-1.23***
	(0.01)	(0.02)	(0.04)	(0.04)	(0.05)	(0.05)	(0.07)	(0.47)
Completed class 10	-0.01*	0.01	-0.12**	-0.09	0.16**	0.07	0.02	-0.96
	(0.01)	(0.02)	(0.05)	(0.05)	(0.07)	(0.06)	(0.08)	(0.59)
Completed class 12	-0.00	0.05**	-0.09	-0.00	0.14	0.14*	0.09	-1.20**
	(0.00)	(0.02)	(0.06)	(0.07)	(0.09)	(0.07)	(0.08)	(0.59)
College and higher	-0.00	0.00	-0.15**	-0.14**	0.22**	0.01	-0.08	-0.63
	(0.00)	(0.03)	(0.06)	(0.07)	(0.09)	(0.09)	(0.14)	(0.73)
Paternal education, years	-0.00	-0.00	0.00	0.01**	-0.00	0.00	0.00	-0.04
	(0.00)	(0.00)	(0.00)	(0.00)	(0.00)	(0.00)	(0.01)	(0.03)
Household size, persons	-0.00	0.00*	-0.01	0.01	-0.01	-0.02*	-0.00	0.03
	(0.00)	(0.00)	(0.01)	(0.01)	(0.01)	(0.01)	(0.01)	(0.08)
**Household SES**:								
Poorest (ref)	-	-	-	-	-	-	-	-
Quintile 2	0.01	0.05**	0.06	0.14***	0.04	0.04	0.06	0.08
	(0.01)	(0.03)	(0.04)	(0.04)	(0.05)	(0.04)	(0.07)	(0.52)
Quintile 3	-0.00	0.06**	0.08*	0.19***	0.04	0.03	0.04	-1.85***
	(0.01)	(0.02)	(0.05)	(0.05)	(0.06)	(0.05)	(0.07)	(0.53)
Quintile 4	0.01	0.06***	0.08	0.18***	0.03	0.00	0.06	-2.71***
	(0.01)	(0.02)	(0.05)	(0.05)	(0.06)	(0.05)	(0.08)	(0.56)
Quintile 5	-0.00	0.05*	0.12**	0.27***	-0.02	0.01	0.12	-4.29***
	(0.01)	(0.02)	(0.06)	(0.05)	(0.07)	(0.06)	(0.08)	(0.63)
**Maternal caste**:								
Scheduled caste (ref)	-	-	-	-	-	-	-	-
Scheduled tribe	0.00	-0.02	-0.08*	-0.00	0.06	-0.03	0.01	-0.12
	(0.01)	(0.02)	(0.04)	(0.04)	(0.06)	(0.04)	(0.07)	(0.59)
OBC	0.00	-0.02*	-0.08**	-0.01	-0.03	-0.05	0.02	-0.78
	(0.01)	(0.01)	(0.04)	(0.04)	(0.05)	(0.04)	(0.06)	(0.54)
General caste	0.01	-0.03**	-0.10*	-0.14***	0.01	-0.05	-0.04	0.68
	(0.01)	(0.01)	(0.05)	(0.05)	(0.07)	(0.05)	(0.07)	(0.78)
Child is a girl	-0.00	0.00	-0.04*	0.00	0.08**	0.01	0.05	
	(0.00)	(0.01)	(0.02)	(0.03)	(0.03)	(0.03)	(0.04)	
Child's age in months	0.00	0.00	-0.00	-0.00	-0.00	0.00	-0.01*	
	(0.00)	(0.00)	(0.00)	(0.00)	(0.00)	(0.00)	(0.01)	
N	1161	1161	1161	1161	927	603	376	1161

*** p<0.01,

** p<0.05,

* p<0.1;

HFIAS = Household Food Insecurity Access Scale; OBC = Other Backward Class; PDS = Public Distribution System; VHND = Village Health and Nutrition Day. Numbers reported are the coefficient on the dummy for treatment, with standard errors in parentheses. Columns 1–4 are for the full sample, column 5 for children over the age of 3 months, column 6 for children over the age of 6 months, and column 7 for children over the age of 12 months. Linear probability models are employed in all cases. Models control for child age, child sex, maternal age, maternal education, paternal education, maternal caste group, participation in the VHND, household SES, household size, and district and block fixed effects. Standard errors are clustered at the level of the block

Women who received money from the *Mamata* scheme were 5 percentage points (pp) more likely to have received ANC services (p<0.01) and 10 pp more likely to have received IFA tablets during pregnancy (p<0.01) (Table 6). However, no significant difference was observed between beneficiary and non-beneficiary women on whether they received postnatal counseling, whether the child was exclusively breastfed, and whether the child was fully immunized.

Participation in the VHND was significantly positively associated with receipt of antenatal services (3 pp, p<0.05), of IFA tablets (5 pp, p<0.05) and of counselling during pregnancy (15 pp, p<0.01). It was also associated with a 6 pp increase in the likelihood that the child was exclusively breastfed.

Compared to the lowest SES quintile, women from higher SES quintiles were more likely to have received ANC services, IFA tablets, and counseling during pregnancy.

Counterintuitively, compared to the women with no education, women with more education were *less likely* to receive IFA tablets from an AWW or ASHA during pregnancy, but more likely to have received postnatal counseling on the duration of breastfeeding.

The results by child gender were also mixed. We observed that being a girl child was associated with a 4 pp decrease in the likelihood of receiving IFA tablets (p<0.1) and an 8 pp increase in the likelihood of receiving postnatal counseling (p<0.05). Other outcomes did not display any significant association with child gender.

Receipt of transfer from the *Mamata* scheme was associated with a 0.84 decrease in the overall HFIAS score (p<0.05) (Table 6). Compared to the mean value of the HFIAS in the full sample (3.44, Table 5) this represented a sizeable decline.

As mentioned above, the PDS provides an in-kind transfer to improve household food security. Table 6 presents the association between the household purchasing something from the PDS and food security, allowing us to compare the effects of an in-kind (PDS food) versus cash transfer (money from the *Mamata* scheme). Purchasing from the PDS was associated with a somewhat larger 1.23 point decrease in the overall food insecurity score (p<0.01), a 7 pp decrease in the domain of anxiety (p<0.05) and an 11 pp decrease in the domain of insufficient quantity (p<0.01).

S2 Table presents the full set of results of the OLS regressions for food security outcomes. We observed significant associations with education and SES–households with higher SES and more educated mothers had higher food security outcomes. In addition, OBC households had higher food security than SC households (the base caste category).

### Nearest-neighbor matching and inverse-probability weighting models

The OLS results presented above are subject to selection bias, which raises concerns about the point estimates reported. To allay these concerns, we present the results of the matching and IPW models for exposure to nutrition interventions and overall household food security (Table 7).

**Table 7:** Nearest-neighbor and IPW estimates of the ATE for exposure to nutrition interventions and household food security.

ATE of receiving *Mamata* money:	Registered pregnancy (1)	Received any antenatal services (2)	Received IFA tablets during pregnancy (3)	Received counselling during pregnancy (4)	Received counselling on duration of breastfeeding (5)	Child was exclusively breastfed (6)	Child is fully immunized (7)	Overall HFIAS score (8)
Nearest neighbor matching	0.01**	0.06***	0.12***	0.07**	-0.02	0.01	0.10**	-1.29***
	(0.00)	(0.01)	(0.03)	(0.03)	(0.04)	(0.03)	(0.05)	(0.29)
IPW	0.01**	0.05***	0.09***	-0.03	-0.05	-0.01	0.09	-1.15***
	(0.00)	(0.01)	(0.03)	(0.06)	(0.04)	(0.03)	(0.06)	(0.33)
N	1161	1161	1161	1161	927	603	376	1161

*** p<0.01,

** p<0.05,

* p<0.1.

HFIAS = Household Food Insecurity Access Scale. Numbers reported are the coefficient on the dummy for treatment, with standard errors in parentheses. Columns 1–4 are for the full sample, column 5 for children over the age of 3 months, column 6 for children over the age of 6 months, and column 7 for children over the age of 12 months. For nearest neighbor matching, units are matched on maternal age, maternal education, paternal education, maternal caste group, household SES, household size, child age and sex, and district and block fixed effects. Standard errors are clustered at the level of the block.

The nearest-neighbor matching estimates of the average treatment effect (ATE) of receiving *Mamata* money show positive impacts on pregnancy registration (1 pp, p<0.05), receiving antenatal services (6 pp, p<0.01), receiving IFA tablets during pregnancy (12 pp e, p<0.01), receiving counselling during pregnancy (7 pp, p<0.05) and on full child immunization (10 pp, p<0.05).

The IPW estimates of the ATE of receiving *Mamata* money are very similar to the nearest-neighbor estimates (Table 7). According to this method, receiving *Mamata* money was associated with a statistically significant increase in the probability of the pregnancy being registered (1 pp, p<0.05), receiving antenatal services (5 pp, p<0.01) and receiving IFA tablets (9 pp, p<0.01).

The matching and IPW estimates of the ATE of receiving *Mamata* money on household food security are very similar to each other and to the OLS estimates presented earlier (Table 7). The nearest-neighbor matching estimate of the effect of receiving *Mamata* money on the overall HFIAS score was a reduction of 1.29 points (p<0.01), compared to 1.15 points when using IPW (p<0.01), and .84 points when using OLS (p<0.05). The full set of results on the 9 sub-components and 3 domains of the HFIAS is presented in S3 Table.

We have presented the OLS results in greater detail for two reasons. First, as shown in Tables 6 and 7, the three methods–OLS, nearest-neighbor matching and IPW–all yield similar estimates of the average treatment effect of receiving money from the *Mamata* scheme, suggesting that the extent of bias in the OLS estimates is not likely to be large. Second, unlike the nearest-neighbor matching and IPW estimations, the OLS results allow us to examine other interesting and, in some cases, ‘counter-intuitive’ associations of the outcome variables with individual and household characteristics.

## Discussion

We investigated the impact of a CCT program on the exposure to essential nutrition interventions and household food security in three select districts in the eastern Indian state of Odisha. The *Mamata* scheme provides mothers with INR 5000 in four installments on fulfilment of certain conditions. The amount of money transferred is a sizeable portion of the annual household income in the state [38]. We observed that the scheme positively affected adherence to certain sets of conditions, especially those during pregnancy. In particular, it increased the likelihood of pregnancy registration and receipt of antenatal services and IFA tablets from government frontline health workers. Interestingly, while there is evidence that household investment in the health of girl children is lower in countries like India that exhibit strong son-preference [43,44], we did not find a significant negative association between health outcomes and the child being female, except for a decline in the likelihood of receiving IFA tablets. Receipt of money under the *Mamata* scheme also positively affected household food security. It was associated with an overall decline in the household’s food insecurity score, as well as a decline in some of the individual indicators, such as someone in the household eating food they didn’t want to eat or eating smaller meals. These results add to the growing evidence on the effectiveness of cash transfer schemes in India, much of which has been focused on the JSY until now. To the best of our knowledge, our study results are the first quantitative estimates of the impact of the *Mamata* scheme.

This paper contributes to several threads of the literature. *First*, it addresses the debate around the impact of cash transfer schemes globally. We showed that the CCT appears to have large and positive impacts on household food security, while simultaneously encouraging adherence to conditions related to health and nutrition service use. This is in line with evidence from other countries that finds that CCTs improve food security and nutrition outcomes [9–20,45]. The association of the receipt of *Mamata* money with increased likelihood of receiving ANC services is particularly encouraging given that ANC is recognized as a health intervention that is particularly inequitably distributed in low- and middle-income countries [30].

*Second*, our study provides some insight into the design aspects of CCTs—the size and regularity of payments, the conditions, and the nature of the recipients. CCTs need to take the local context and constraints into consideration in order to be effective [46]. Unless the cash transfer is sufficiently large and regular, the impacts are likely to be small [19,34,47]. We observed that the effect of the scheme on conditions tapered off after the first installment was paid, which could be the result of delayed or incorrect second and third tranche payments. Another possibility is the increased complexity and requirement for documentation of later conditions, which could be acting as a deterrent [24,28,33]. In populations as poor and poorly-educated as our study sample, conditions that are easy to fulfil and verify could improve adherence further. Complicated documentation can also contribute to the exclusion of those who are arguably in greatest need of this transfer [34]. In fact, cash transfer schemes in India have been shown to exclude poor, less educated, landless and lower caste women for this reason [24,25,27,33]. Our study results indicated a similar exclusion; women of higher SES were more likely to receive *Mamata* money compared to those of lower SES, and SES was significantly and positively associated with the exposure to nutrition interventions.

In contrast to the evidence of imperfect targeting of the *Mamata* scheme, we also found that women with more education were less likely to receive IFA tablets from a government health worker (ASHA or AWW) during pregnancy, but more likely to have received post-natal counseling on the duration of breastfeeding. To the extent that wealth and educational attainment are correlated, this result appears to be counter to the SES-related results just discussed. One explanation is that more educated women are more likely to access private healthcare, which is generally considered to be of better quality than publicly provided healthcare, and are receiving their IFA tablets from private sources. Since we do not specify the source of the postnatal counseling, it is possible that for women with greater education this counseling is also being provided by private healthcare professionals.

*Third*, it provides a comparison of the impacts on food security of an in-kind transfer (from the PDS) to the *Mamata* cash transfer. While we cannot make a meaningful cost-benefit comparison of the two without information on what was purchased, our results show that the effects of purchasing food items from the PDS were larger in magnitude. This finding is important given the existing mixed evidence on the effectiveness of the food subsidies.

In Odisha, the PDS program has been documented as functioning better and with fewer incidents of corruption than in several other Indian states [48], so the association between PDS and household food insecurity in Odisha is not necessarily generalizable. Odisha is a state that primarily consumes rice and has high levels of poverty, making it a good candidate for a universal PDS. However, a universal PDS setting may not be extendable to a state like Uttar Pradesh which produces (in surplus) and consumes both rice and wheat and therefore would require a greater check on pilferage, or to states like Punjab and Haryana where poverty is significantly lower, resulting in low demand for PDS cereal. Features such as these make it harder to emulate successes in the PDS than in CCTs, where the ‘commodity’ being distributed–cash–is inherently homogenous and fungible.

There is some evidence that the PDS is “self-targeting” towards poor households [49], and conditional cash transfers towards better educated households [24,25,28,33]. However, the differences in targeting between cash and in-kind transfers relate to implementation challenges and the use of technology to facilitate improvements in uptake. For example, successful identification and issuance of ration cards to poor households doesn’t necessarily guarantee uptake. In opaque systems, the PDS is infamous for leakage and denial of food grains to the poor [50].

*Fourth*, our study demonstrates several important outcomes associated with CCTs implemented at scale in India, particularly where a well-functioning health system exist. For CCTs to have an impact, it has to be feasible for households or individuals to meet the conditions, which requires the availability of and access to adequate services. Odisha is a good state to test such a scheme in, as it has a strong and relatively equitable healthcare system [36]. However, even within Odisha we see differences that might be driven in part by differences in service delivery. Jagatsinghpur, the better performing district in our sample based on the criteria of service delivery and household factors, also has the highest proportion of *Mamata* beneficiaries (Table 1). This is suggestive of the importance of stronger service provision to support the functioning of a CCT.

Our *fifth* and final point relates to the importance of behavior change communication (BCC) to achieve and sustain impacts on behaviors linked with the CCTs [22]. At present, delivery of nutrition or health-related BCC such as interpersonal counseling is weak [51]. The significant impact of *Mamata* scheme money on receipt of ANC and IFA tablets from government frontline health workers and its lack of impact on exclusive breastfeeding indicates the need to strengthen provision of BCC. Exclusive breastfeeding is likely to be driven more by individual and household-level factors that are outside the purview of the delivery system, which might explain why we do not find a significant impact of *Mamata* money on this outcome. In the context of rural India, the VHND is an important platform for delivering nutrition BCC interventions, and we observed that participation in the VHND is indeed positively associated with receipt of counseling during pregnancy and with exclusive breastfeeding. Further research is needed to untangle the effect of this delivery platform from that of the cash transfers.

Some limitations to our study includes its geographical focus; our study sample was not designed to be representative of Odisha. It is a single cross-sectional survey administered after the introduction of the scheme, and beneficiaries were not randomized into treatment and control groups. Indeed, we show that the two groups differ, with beneficiaries being on average wealthier and more educated, a finding that also speaks also to the imperfect targeting of the scheme. We addressed the concern of selection bias in the analysis by presenting estimates from OLS regressions, nearest-neighbor matching models and inverse-probability weighting models. Reassuringly, our results are consistent across all three models. These results can be viewed as indicative of the impacts of the CCT in our study context.

## Conclusions

This paper provides the first quantitative estimates of the impact of the *Mamata* maternity benefit scheme on exposure to nutrition interventions and household food security in Odisha. We show that the scheme had positive and significant effects on food security, registration of the pregnancy and receipt of ANC and of IFA tablets from government frontline health workers. However, we caution that without the presence of a strong health system, the fulfillment of conditions may not be as straightforward. Once data from the fourth round of the National Family Health Survey (NFHS-4) become available, further research can be conducted to examine the extent to which improvements in Odisha’s nutrition outcomes have been driven by participation in the *Mamata* scheme.

India continues to contribute substantially to the global burden of maternal and child malnutrition. However, the Prime Minister’s 2017 Union budgetary announcement to scale up maternity benefits nationwide will constitute one of the largest cash transfer schemes in the world and will have the potential to influence several maternal and child health and nutrition indicators. Therefore, the evidence we present here on the impact of a CCT in one state in India is both timely and relevant.

## Supporting information

S1 Table List of assets used in the construction of household socio-economic status. (DOCX)

S2 Table OLS regressions on household food insecurity (full results). (DOCX)

S3 Table Nearest-neighbor and IPW estimates of the ATE for household food security (full results). (DOCX)

S1 Fig Kernel densities of the receipt of *Mamata* money by the propensity score. Source: Author’s calculations.
(DOCX)
