# Supplementary material for: Can conditional cash transfers improve the uptake of nutrition interventions and household food security? Evidence from Odisha’s Mamata scheme
Source: PLoS One. 2017 Dec 11;12(12):e0188952. doi: 10.1371/journal.pone.0188952 (PMC5724821; doi:10.1371/journal.pone.0188952)
Supplement: S1 Table — (DOCX) [file pone.0188952.s001.docx]

**S1 Table: List of assets used in the construction of household socio-economic status**

| **Domain** | **Description of measure used** |
| --- | --- |
| Asset ownership | Total number of durable assets from among the following:   - Stove/gas burner - Refrigerator - Mattress - Pressure cooker - Table - Chair - Electric fan - Radio - Audio cassette/CD player - Television - DVD player - Wall clock/watch - Sewing machine - Bicycle - Cycle rickshaw - Van (tricycle van) - Boat/canoe - Motorcycle/scooter - Mobile phone - Landline phone - Computer/laptop - Bullock cart/horse cart - Thresher - Hand tube well/rower pump - Cart - Water pump - Tractor |
| Livestock ownership | 4 categories, based on number of livestock units (cows, buffaloes, goats, chickens, pigs and others):   - No livestock - Between 1 and 5 units - Between 6 and 10 units - Between 11 and 15 units - More than 16 units |
| Home ownership | Dummy for the household owning both the house and the land it is on |
| Land ownership | Dummy for the household owning any land other than what the house is on |
| Toilet ownership | Dummy for the household having a toilet facility in the household |
