# Supplementary material for: Can conditional cash transfers improve the uptake of nutrition interventions and household food security? Evidence from Odisha’s Mamata scheme
Source: PLoS One. 2017 Dec 11;12(12):e0188952. doi: 10.1371/journal.pone.0188952 (PMC5724821; doi:10.1371/journal.pone.0188952)
Supplement: S2 Table — (DOCX) [file pone.0188952.s002.docx]

**S2 Table: OLS regressions on household food insecurity (full results)**

|  | **HFIAS score**  **(1-27)** | **Worries HH won't have enough food** | **Not able to eat preferred foods** | **Eats only few varieties of food** | **Ate food didn't want to eat** | **Ate smaller meals** | **Ate fewer meals** | **No food in house** | **Slept hungry at night** | **Did not eat for 24 hours** | **Anxiety** | **Insufficient quality** | **Insufficient quantity** |
| --- | --- | --- | --- | --- | --- | --- | --- | --- | --- | --- | --- | --- | --- |
| Received money from *Mamata* | -0.84** | -0.03 | -0.00 | -0.07** | -0.08** | -0.08*** | -0.07** | -0.06** | -0.05** | -0.04* | -0.03 | -0.00 | -0.05 |
|  | (0.36) | (0.03) | (0.03) | (0.03) | (0.03) | (0.03) | (0.03) | (0.03) | (0.02) | (0.02) | (0.03) | (0.03) | (0.03) |
| Bought from PDS | -1.23*** | -0.07** | -0.00 | -0.08*** | -0.07*** | -0.10*** | -0.05* | -0.05* | -0.03 | -0.03 | -0.07** | -0.02 | -0.11*** |
|  | (0.36) | (0.03) | (0.03) | (0.03) | (0.03) | (0.03) | (0.03) | (0.02) | (0.02) | (0.02) | (0.03) | (0.03) | (0.03) |
| Maternal age | -0.02 | 0.00 | 0.01*** | 0.00 | -0.00 | -0.00 | -0.00 | -0.00 | -0.00 | -0.00 | 0.00 | 0.01** | -0.01* |
|  | (0.04) | (0.00) | (0.00) | (0.00) | (0.00) | (0.00) | (0.00) | (0.00) | (0.00) | (0.00) | (0.00) | (0.00) | (0.00) |
| Maternal education: | | | | | | | | | | | | | |
| No education (ref) | - | - | - | - | - | - | - | - | - | - | - | - | - |
| Primary school | 0.18 | -0.05 | -0.09* | 0.03 | 0.01 | 0.08 | 0.00 | 0.02 | -0.03 | -0.01 | -0.05 | -0.11** | -0.01 |
|  | (0.58) | (0.05) | (0.05) | (0.05) | (0.05) | (0.05) | (0.05) | (0.05) | (0.04) | (0.04) | (0.05) | (0.05) | (0.05) |
| Middle school | -1.23*** | -0.12** | -0.19*** | -0.09* | -0.06 | -0.01 | -0.06 | -0.06 | -0.07* | -0.09** | -0.12** | -0.16*** | -0.06 |
|  | (0.47) | (0.05) | (0.05) | (0.04) | (0.05) | (0.04) | (0.04) | (0.04) | (0.04) | (0.04) | (0.05) | (0.05) | (0.05) |
| Completed class 10 | -0.96 | -0.10* | -0.14** | -0.07 | -0.08 | 0.01 | -0.06 | -0.07 | -0.06 | -0.07* | -0.10* | -0.15*** | -0.06 |
|  | (0.59) | (0.06) | (0.06) | (0.05) | (0.06) | (0.05) | (0.05) | (0.04) | (0.04) | (0.04) | (0.06) | (0.06) | (0.05) |
| Completed class 12 | -1.20** | -0.06 | -0.19** | -0.10* | -0.09 | -0.01 | -0.04 | -0.07 | -0.06 | -0.03 | -0.06 | -0.18** | -0.06 |
|  | (0.59) | (0.07) | (0.07) | (0.06) | (0.06) | (0.05) | (0.05) | (0.05) | (0.04) | (0.04) | (0.07) | (0.07) | (0.06) |
| College and higher | -0.63 | -0.08 | -0.17** | -0.03 | -0.00 | 0.04 | -0.01 | -0.06 | -0.10** | -0.10*** | -0.08 | -0.17** | -0.05 |
|  | (0.73) | (0.08) | (0.07) | (0.07) | (0.07) | (0.06) | (0.06) | (0.05) | (0.04) | (0.04) | (0.08) | (0.07) | (0.06) |
| Paternal education | -0.04 | 0.00 | 0.00 | -0.00 | -0.00 | -0.00** | -0.00* | -0.01** | -0.00** | -0.00** | 0.00 | -0.00 | -0.00* |
|  | (0.03) | (0.00) | (0.00) | (0.00) | (0.00) | (0.00) | (0.00) | (0.00) | (0.00) | (0.00) | (0.00) | (0.00) | (0.00) |
| Household size | 0.03 | -0.01 | 0.00 | 0.00 | 0.01 | 0.01* | 0.00 | 0.00 | 0.00 | -0.00 | -0.01 | 0.00 | 0.01* |
|  | (0.08) | (0.01) | (0.01) | (0.01) | (0.01) | (0.01) | (0.01) | (0.01) | (0.00) | (0.00) | (0.01) | (0.01) | (0.01) |
| Household SES: | | | | | | | | | | | | | |
| Poorest (ref) | - | - | - | - | - | - | - | - | - | - | - | - | - |
| Quintile 2 | 0.08 | -0.03 | 0.07 | -0.00 | -0.05 | -0.00 | -0.02 | -0.03 | -0.08* | -0.06 | -0.03 | -0.05 | -0.10** |
|  | (0.52) | (0.05) | (0.05) | (0.05) | (0.05) | (0.05) | (0.04) | (0.04) | (0.04) | (0.04) | (0.05) | (0.04) | (0.04) |
| Quintile 3 | -1.85*** | -0.24*** | 0.02 | -0.13*** | -0.17*** | -0.18*** | -0.17*** | -0.10** | -0.17*** | -0.14*** | -0.24*** | -0.15*** | -0.33*** |
|  | (0.53) | (0.06) | (0.05) | (0.05) | (0.05) | (0.05) | (0.04) | (0.04) | (0.04) | (0.04) | (0.06) | (0.05) | (0.05) |
| Quintile 4 | -2.71*** | -0.25*** | -0.12** | -0.20*** | -0.20*** | -0.20*** | -0.20*** | -0.09** | -0.18*** | -0.15*** | -0.25*** | -0.31*** | -0.36*** |
|  | (0.56) | (0.06) | (0.06) | (0.05) | (0.05) | (0.05) | (0.05) | (0.05) | (0.04) | (0.04) | (0.06) | (0.05) | (0.05) |
| Quintile 5 | -4.29*** | -0.41*** | -0.24*** | -0.33*** | -0.33*** | -0.31*** | -0.30*** | -0.16*** | -0.22*** | -0.20*** | -0.41*** | -0.46*** | -0.50*** |
|  | (0.63) | (0.06) | (0.06) | (0.06) | (0.06) | (0.05) | (0.05) | (0.05) | (0.04) | (0.04) | (0.06) | (0.06) | (0.06) |
| Scheduled caste (ref) | - | - | - | - | - | - | - | - | - | - | - | - | - |
| Scheduled tribe | -0.12 | -0.06 | -0.06 | -0.01 | -0.02 | -0.02 | 0.01 | 0.01 | -0.01 | -0.01 | -0.06 | 0.01 | -0.00 |
|  | (0.59) | (0.05) | (0.06) | (0.05) | (0.05) | (0.05) | (0.04) | (0.04) | (0.03) | (0.03) | (0.05) | (0.05) | (0.05) |
| OBC | -0.78 | -0.12*** | -0.11** | -0.10** | -0.07 | -0.07* | -0.05 | -0.04 | -0.03 | 0.00 | -0.12*** | -0.08* | -0.07 |
|  | (0.54) | (0.05) | (0.05) | (0.04) | (0.04) | (0.04) | (0.04) | (0.04) | (0.03) | (0.03) | (0.05) | (0.05) | (0.04) |
| General caste | 0.68 | -0.03 | -0.08 | 0.03 | 0.01 | 0.06 | 0.05 | 0.02 | 0.01 | 0.05 | -0.03 | -0.05 | 0.09 |
|  | (0.78) | (0.06) | (0.06) | (0.06) | (0.06) | (0.06) | (0.06) | (0.05) | (0.04) | (0.05) | (0.06) | (0.06) | (0.06) |
| constant | 7.99*** | 0.71*** | 0.38** | 0.55*** | 0.74*** | 0.47*** | 0.48*** | 0.41*** | 0.47*** | 0.37*** | 0.71*** | 0.69*** | 0.81*** |
|  | (1.55) | (0.14) | (0.15) | (0.13) | (0.14) | (0.13) | (0.12) | (0.10) | (0.10) | (0.10) | (0.14) | (0.14) | (0.14) |
| N | 1161 | 1161 | 1161 | 1161 | 1161 | 1161 | 1161 | 1161 | 1161 | 1161 | 1161 | 1161 | 1161 |

Notes: *** p<0.01, ** p<0.05, * p<0.1. Numbers reported are the coefficient on the dummy for treatment, with standard errors in parentheses. Linear probability models are employed in all cases. Models control for child age, child sex, maternal age, maternal education, paternal education, maternal caste group, participation in the VHND, household SES, household size, and district and block fixed effects. Standard errors are clustered at the level of the block.
