# Supplementary material for: Can conditional cash transfers improve the uptake of nutrition interventions and household food security? Evidence from Odisha’s Mamata scheme
Source: PLoS One. 2017 Dec 11;12(12):e0188952. doi: 10.1371/journal.pone.0188952 (PMC5724821; doi:10.1371/journal.pone.0188952)
Supplement: S3 Table — (DOCX) [file pone.0188952.s003.docx]

**S3 Table:** Nearest-neighbor and IPW estimates of the ATE for household food security (full results)

Notes: *** p<0.01, ** p<0.05, * p<0.1.Numbers reported are the coefficient on the dummy for treatment, with standard errors in parentheses. For nearest neighbor matching, units are matched on maternal age, education and caste group, paternal, father’s education, , household SES, household size, whether the household purchased something from the PDS in the 30 days prior, and district and block fixed effects. Standard errors are clustered at the level of the block.

| ATE of receiving *Mamata* money | **HFIAS score (1-27)** | **Worries HH won't have enough food** | **Not able to eat preferred foods** | **Eats only few varieties of food** | **Ate food didn't want to eat** | **Ate smaller meals** | **Ate fewer meals** | **No food in house** | **Slept hungry at night** | **Did not eat for 24 hours** | **Anxiety** | **Insufficient quality** | **Insufficient quantity** |
| --- | --- | --- | --- | --- | --- | --- | --- | --- | --- | --- | --- | --- | --- |
| Nearest neighbor matching | -1.29*** | -0.07** | -0.04 | -0.11*** | -0.10*** | -0.11*** | -0.10*** | -0.08*** | -0.08*** | -0.07*** | -0.07** | -0.04 | -0.09*** |
|  | (0.29) | (0.03) | (0.03) | (0.03) | (0.03) | (0.03) | (0.02) | (0.02) | (0.02) | (0.02) | (0.03) | (0.03) | (0.03) |
| IPW | -1.15*** | -0.06* | -0.02 | -0.09*** | -0.09*** | -0.11*** | -0.09*** | -0.07*** | -0.07*** | -0.06** | -0.06* | -0.01 | -0.07** |
|  | (0.33) | (0.03) | (0.03) | (0.03) | (0.03) | (0.03) | (0.03) | (0.03) | (0.03) | (0.02) | (0.03) | (0.03) | (0.03) |
| N | 1161 | 1161 | 1161 | 1161 | 1161 | 1161 | 1161 | 1161 | 1161 | 1161 | 1161 | 1161 | 1161 |
