# Supplementary material for: Can conditional cash transfers improve the uptake of nutrition interventions and household food security? Evidence from Odisha’s Mamata scheme
Source: PLoS One. 2017 Dec 11;12(12):e0188952. doi: 10.1371/journal.pone.0188952 (PMC5724821; doi:10.1371/journal.pone.0188952)
Supplement: S1 Fig — Source: Author’s calculations. (DOCX) [file pone.0188952.s004.docx]

**S1 Figure: Kernel densities of the receipt of Mamata money by the propensity score**

Source: Author’s calculations.
